# A rare case of metastatic super giant basal cell carcinoma of the thoracoabdominal wall

**DOI:** 10.1016/j.jdcr.2025.10.009

**Published:** 2025-10-11

**Authors:** Dong Eun Lee, Matthew D. Wynne, Nicholas J. Collier, Lynne A. Jamieson, Donna M. Cummins

**Affiliations:** a The Dermatology Centre, Salford Royal Hospital, Northern Care Alliance NHS Foundation Trust, Manchester, United Kingdom; b Division of Musculoskeletal and Dermatological Sciences, Centre for Dermatology Research, The University of Manchester, Manchester, United Kingdom; c Department of Dermatology, Beaumont Hospital, Dublin, Ireland; d Department of Medicine, Royal College of Surgeons in Ireland, Dublin, Ireland; e Department of Cellular Pathology, Salford Royal Hospital, Northern Care Alliance NHS Foundation Trust, Manchester, United Kingdom

**Keywords:** basal cell carcinoma, giant basal cell carcinoma, metastatic basal cell carcinoma, super giant basal cell carcinoma

## Introduction

Basal cell carcinoma (BCC) is the most prevalent form of cutaneous malignancy, originating from the epidermal basal layer.^1^ It typically exhibits a slow-growing and locally invasive nature. The primary risk factor is exposure to ultraviolet radiation, which accounts for the higher prevalence of BCCs in sun-exposed areas like the face or scalp.^2^ While metastasis is uncommon in BCC, it can cause significant local tissue destruction, especially when neglected. This highlights the importance of timely diagnosis and treatment. Giant BCC is a rare subtype, defined by a tumor measuring over 5 cm in diameter. An even rarer variant is super-giant basal cell carcinoma (SGBCC), which has a diameter larger than 20 cm.^2^^,^^3^ This case report describes a 49-year-old patient with a metastatic SGBCC.

## Case presentation

A 49-year-old Caucasian male presented to the emergency department with breathlessness. The patient had no known medical history. He had lived in Thailand for the past decade, working as an English teacher, with only brief visits to the United Kingdom. He planned to return to Thailand soon, but worsening symptoms and family concern led him to seek medical care.

**Fig 1:**
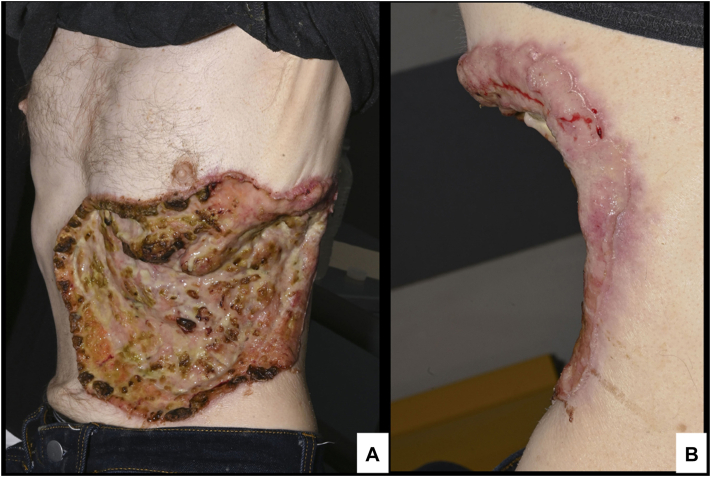
**A,** A 25 × 16 cm basal cell carcinoma with extensive, ulcerated appearance with underlying ribs exposed - anterolateral perspective. **B,** From posterior perspective to demonstrate the friable, rolled edges of the lesion.

Physical examination revealed a large fungating lesion (25 × 16 cm) in the left thoracoabdominal region, exposing parts of the underlying ribs [Fig 1]. The lesion appeared sloughy with pink rolled edges. The patient reported having initially noticed a small, wart-like growth beneath his left lower rib approximately a decade prior. The lesion gradually increased in size until 18 months ago, at which point the growth accelerated. He also experienced approximately 20 kilograms of weight loss. Due to the fear of diagnosis, the patient did not seek medical care.

**Fig 2:**
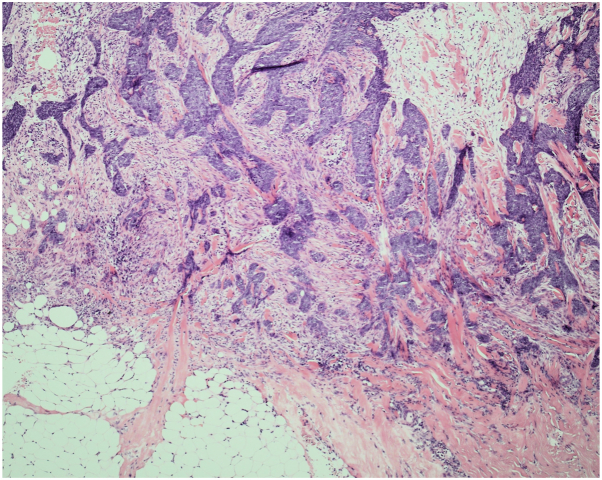
Infiltrative basal cell carcinoma infiltrating into the subcutaneous fat at the deep aspect of the specimen. Tissues stained with hematoxylin and eosin stain.

**Fig 3:**
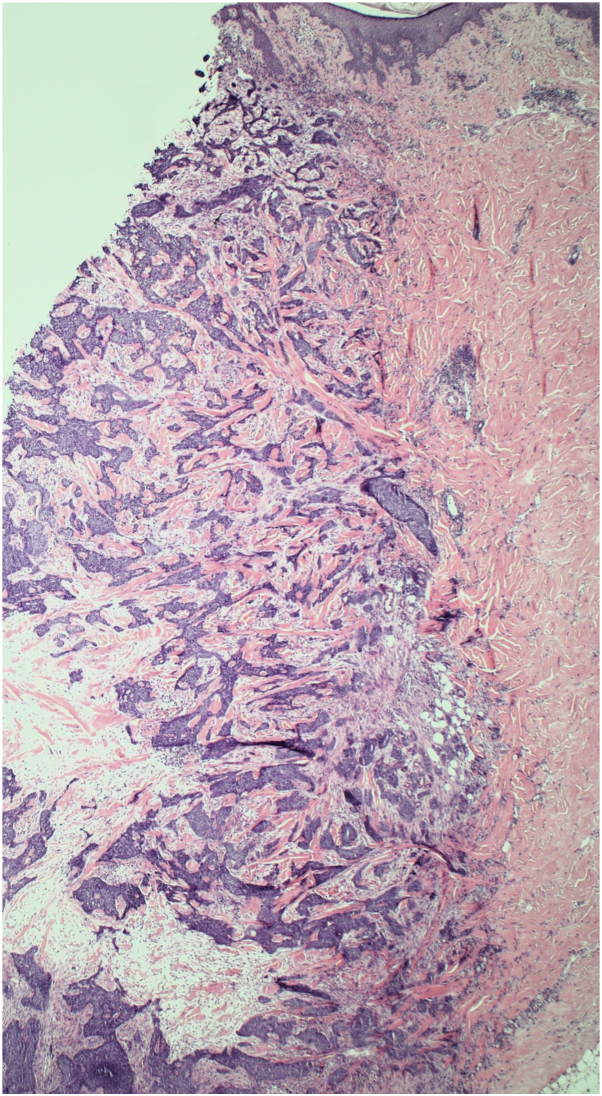
Infiltrative basal cell carcinoma infiltrating through the full thickness of the dermis and into the subcutaneous fat to the deep aspect of the sample. Tissues stained with hematoxylin and eosin stain. In this sample, the thickness of the tumor extension was at least 9 mm.

Initial investigations revealed a hemoglobin level of 43 g/dL, likely the primary cause of breathlessness. Infectious disease serology was negative. A computed tomography scan confirmed a large, ulcerating tumor invading into the left thoracic cavity and a small right apical pneumothorax, which was managed conservatively. Given the severe iron deficiency anemia, likely secondary to ulceration and chronic bleeding, the patient received a transfusion of 5 units of blood. He was evaluated by the dermatology team and incisional biopsies confirmed the diagnosis of nodular and infiltrative BCC [Figs 2 and 3]. The lesion size and associated morbidity were consistent with SGBCC.^2^

**Fig 4:**
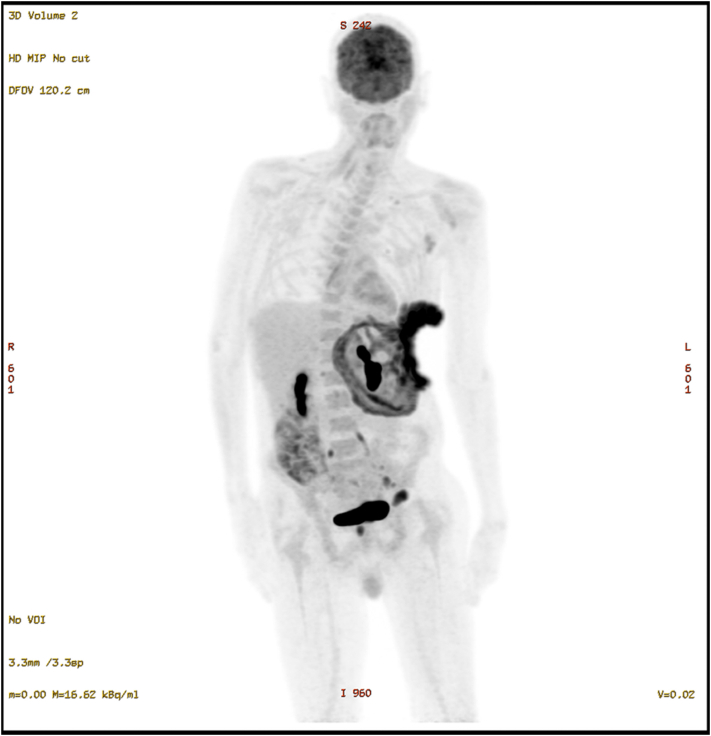
Fludeoxyglucose-18 positron emission tomography-computed tomography (FDG PET-CT) scan image showing ulcerative left lower chest/upper abdomen cutaneous thickening with avid uptake concerning for malignancy and distant metastases in lymph nodes, liver and bones.

The patient was urgently referred to the plastic surgery and specialist skin cancer multidisciplinary teams. However, due to the extensive nature of the lesion, the complexity of the surgery, and the high risk of complications, surgical reconstruction was deemed unsuitable. Furthermore, the multidisciplinary team's review of the previous imaging revealed a suspicious left axillary lymph node, raising concern for metastasis. The patient was further referred to medical oncology for consideration of neoadjuvant vismodegib therapy, with the goal of reducing tumor size for surgical excision. A whole-body PET scan and fine-needle aspiration of the lymph node were also arranged [Fig 4].

Unfortunately, these investigations revealed metastases to the liver, bones, and lymph nodes. Although treatment options of vismodegib and cemiplimab were carefully evaluated, the multidisciplinary team recognized that rapid tumor shrinkage could create an unstable, nonreconstructable abdominal wall defect, with risk of life-threatening infection and impaired quality of life. Ultimately, the patient understood these risks and elected to a conservative, symptom-focused approach. Palliative care was therefore initiated. This case underscores the challenges in managing SGBCC, particularly when diagnosis is delayed, leading to advanced disease and limited therapeutic options.

## Discussion

SGBCC is a rare and aggressive variant of BCC, characterized by exceptionally large size, exceeding 20 cm in diameter. The literature reports approximately 21 cases of SGBCC.^2^^,^^4^ Risk factors include low socioeconomic status, physical or mental disability, healthcare avoidance, and self-neglect. On average, the time between onset and diagnosis is approximately 10 years. Unlike typical BCCs that occur on sun-exposed sites, SGBCCs often appear in areas hidden by clothing, like the trunk and limbs, contributing to neglect and delayed recognition.^2^

While metastasis is exceedingly rare, occurring in 0.03% of cases, the likelihood of metastasis and associated mortality increases with tumor size. Lesions exceeding 10 cm in diameter demonstrate about 50% increased risk of metastasis.^2^ Prognosis is poor with distant metastasis, with median survival of 24 months compared with 87 months for regional metastasis.^5^

Iron deficiency anemia has been described in giant BCCs, although reported cases are rare. Tumor friability and ulceration make chronic pinpoint bleeding the most likely mechanism. Myelophthisic anemia secondary to marrow infiltration has also been reported but is exceptionally uncommon.^4^ In our case, the anemia was most likely due to tumor-related bleeding, with possible contribution from poor diet and self-neglect.

There is no consistent guideline for SGBCC due to its rarity. However, surgical excision remains the gold standard treatment for BCC, regardless of size and location. For low-risk BCCs, a surgical margin of 4 mm is typically recommended, while larger BCCs may require 13 mm or more.^2^ Mohs micrographic surgery, allowing precise intraoperative mapping of the entire surgical margin, has been recommended.^6^

For unresectable, locally advanced, or metastatic BCCs, Hedgehog pathway inhibitors such as vismodegib and sonidegib have demonstrated efficacy in downstaging disease permitting surgery.^7^ Vismodegib inhibits the Hedgehog signaling pathway by targeting the Smoothened protein, a key transmembrane protein in signal transduction pathway, thereby suppressing the pathway's activity.^8^ Cemiplimab, an anti-programmed cell death protein-1 monoclonal antibody, has demonstrated clinically meaningful activity with an acceptable safety profile in patients with locally advanced BCC refractory to Hedgehog inhibitor therapy.^9^

The present case highlights the potential impact of SGBCCs, particularly those with distant metastases. Despite reports of successful outcomes with surgical excision with neoadjuvant vismodegib or with cemiplimab, these options were not feasible for our patient. Our case illustrates a rare presentation of an inoperable and medically intractable SGBCC in a relatively young and otherwise healthy individual. This case demonstrates the importance of early diagnosis and intervention for larger, high-risk BCCs, especially in patients with relevant risk factors.

## Conflicts of interest

None disclosed.
